# Hexavalent chromium reduction by chromate-resistant haloalkaliphilic *Halomonas* sp. M-Cr newly isolated from tannery effluent

**DOI:** 10.1080/13102818.2014.937092

**Published:** 2014-10-17

**Authors:** Mona E.M. Mabrouk, Mervat A. Arayes, Soraya A. Sabry

**Affiliations:** ^a^ Botany Department, Faculty of Science, Damanhour University, Damanhour, Egypt; ^b^ Botany and Microbiology Department, Faculty of Science, Alexandria University, Alexandria, Egypt

**Keywords:** bioremediation, Cr(VI) reduction, *Halomonas* sp. M-Cr, haloalkaliphilic, scanning electron microscopy, Plackett–Burman design

## Abstract

The current study aimed to isolate and characterize a chromate-resistant bacterium from tannery effluent, able to reduce Cr(VI) aerobically at high pH and salinity. Environmental contamination by hexavalent chromium, Cr(VI), presents a serious public health problem. Enrichment led to the isolation of 12 bacteria displaying different degrees of chromate reduction. Phenotypic characterization and phylogenetic analysis based on 16S rDNA sequence comparison indicated that the most potent strain belonged to the genus *Halomonas*. The new strain designated as *Halomonas* sp. M-Cr was able to reduce 82% of 50 mg L^−1^ Cr(VI) in 48 h, concomitant with discolouring of yellow colour of the medium and formation of white insoluble precipitate of Cr(III). It exhibited growth up to 3500 mg L^−1^ Cr(VI), 20% NaCl and showed strong Cr(VI) reduction under alkaline condition, pH 10. Scanning electron microscopy revealed precipitation of chromium hydroxide on bacterial cell surfaces, which showed characteristic peak of chromium in energy-dispersive X-ray analysis. Plackett–Burman design was used to evaluate the influence of related parameters for enhancing Cr(VI) reduction. Glucose, yeast extract and KH_2_PO**_4_** were confirmed as significant variables in the medium. Data suggest *Halomonas* sp. M-Cr as a promising candidate for bioremediation of Cr(VI) contaminated effluents particularly in saline and alkaline environments. Up to our knowledge, this is the first report on isolation of haloalkaliphilic *Halomonas* sp. from tannery effluent.

## Introduction

Chromium (Cr) is a toxic heavy metal extensively used in a variety of industrial processes, owing to its impressive corrosion resistance.[1] Cr(VI) containing wastewater has become a well-recognized hazard in water pollution control. Soluble Cr(VI) is extremely toxic and shows carcinogenic and mutagenic effect on biological systems due to its strong oxidizing nature.[2] In contrast, Cr(III) being sparingly soluble, less toxic and bioavailable in comparison to Cr(VI), readily forms insoluble oxides and hydroxides above pH 5.[3] Thus, biotransformation of Cr(VI) to less toxic Cr(III) is an effective strategy for the remediation of Cr(VI) pollution worldwide.[4] The process has been demonstrated in several bacterial species under both aerobic and anaerobic conditions.[5–7]

Leather tanning is an environmentally challenging process and is one of the leading foreign exchanges earning industries in Egypt. Million litres of wastewater from tanneries containing a high amount of Cr(VI) are discharged into the sewage drains and ponds without any treatment. Therefore, in this paper we report the isolation and characterization of a chromate-resistant bacterium from tannery effluent able to reduce Cr(VI) aerobically at high pH and salinity. Statistical optimization of process parameters that enhance reduction of Cr(VI) was also performed. Up to our knowledge this is the first report about chromate (VI) reduction by haloalkaliphilic *Halomonas* sp. isolated from tannery effluent.

## Materials and methods

### Sampling

Samples were collected from different stages of the tanning process (El-Halafawy Leather Tanning Company, Damanhour, EL-Bahera, Egypt) using screw capped sterilized glass bottles, maintained at 4 °C and immediately transported to the laboratory.

### Enrichment and isolation of chromate detoxifying alkaliphilic bacteria

One mL from each sample was enriched in 250 mL Erlenmeyer flasks containing 50 mL of Luria-Bertani (LB) medium (g L^−1^) tryptone 10, yeast extract 5, NaCl 5, supplemented with 50 mg L^−1^ Cr(VI) in the form of K_2_CrO_4_ and adjusted to pH 10 with sodium carbonate. The inoculated flasks were incubated at 30 °C for 72 h in a rotary shaker at 120 rpm, serving as the initial enrichment culture. Subsequent enrichment transfer cultures were established using 5 mL as inoculum. From flasks showing turbidity and colour change from yellow to turbid white,[8] 100 μL aliquots were spread on LB agar plates amended with the same Cr(VI) concentration and incubated at 30 °C for 48 h. Bacterial colonies showing distinct morphologies were selected, purified and preserved at 4 °C or in 30% (V/V%) sterile glycerol.

### Identification of the bacterial strain

The basic biochemical and physiological properties of M-Cr isolate were analysed according to Bergey's Manual of Determinative Bacteriology.[9] Cell morphology was examined by scanning electron microscope (JEOL JEM-5300).

### Molecular characterization

Molecular characterization of the isolate was done by 16S rDNA sequence analysis. DNA was isolated from M-Cr cells using standard procedures.[10] The purity of the isolated DNA was confirmed by gel electrophoresis. Amplification of 16S rDNA gene was performed as previously reported [11] using F 5′AGAGTTTGATCMTGGCTCAG3′ and R 5′TACGGYTACCTTGTTACGACTT3′ as forward and reverse primers. The polymerase chain reaction (PCR) amplification products were analysed by electrophoresis on a 1% agarose gel and purified. An amplified product of 16S rDNA was sequenced using an ABI PRISM 377 DNA Sequencer and ABI PRISM Big Dye Terminator Cycle Sequencing (Perkin Elmer). The 16S rDNA sequence was uploaded to NCBI database using BLASTN program (http://www.ncbi.nlm.nih.gov/blast/; version 2.0) and compared with sequences available in the GenBank database. Sequences of most close members were aligned using CLUSTALW program (http://www.ebi.ac.uk/clustalw). A phylogenetic tree was constructed, using the phylogeny inference package (PHYLIP; version 3.6).

### Chromate reduction by *Halomonas* sp. M-Cr

A seed culture was prepared by transferring a loopfull of 48 h old slant into 25 mL LB media without chromate, pH 10 and incubated aerobically at 30 °C, by shaking at 120 rpm until O.D._600_ of 1.0. Reduction of chromium by *Halomonas* sp. M-Cr was examined by inoculating 25 mL/100 mL flask with 0.5 mL of seed culture. Sterile inoculated broth without Cr(VI) served as the biotic control, and the uninoculated broth with Cr(VI) served as the abiotic control. The biotic control was used to compare the growth of bacteria with or without Cr(VI) and the abiotic control was used to test if any change in Cr(VI) appeared as a result from the presence of the media components. All the cultures including blanks were incubated at 30 °C with continuous shaking (120 rpm). Samples were collected under sterile conditions at regular time intervals to monitor Cr(VI) reduction as well as growth. All experiments were performed in duplicate and mean values were recorded.

### Quantification of growth

Growth of *Halomonas* sp. M-Cr was determined according to Ibrahim et al. [5] by measuring absorbance at 600 nm against distilled water as blank.

### Estimation of hexavalent chromium

The concentration of residual hexavalent chromium was determined spectrophotometrically in the culture supernatant after centrifugation at 10,000 rpm for 10 min at 4 °C to remove any suspended biomass, and assayed at 540 nm using 1,5-diphenylcarbazide (DPC) method.[12]

### Effect of pH and salinity on Cr(VI) reduction

The influence of pH on bacterial growth and chromate reduction was examined by adjusting pH of medium to values ranging from 6 to 11 with predetermined amounts of filter-sterilized (0.22 μm) 1M Na_2_CO_3_ or 1M HCl. The effect of salt concentration was examined by adding different concentrations (50–200 g L^−1^) of NaCl.

### Scanning electron microscopy (SEM) and SEM-EDX analysis of *Halomonas* sp. M-Cr cells

Bacterial cells grown in liquid media with and without Cr(VI) were harvested by centrifugation at 10 000 rpm for 10 min at 4 °C. Cells were fixed, dehydrated and dried using the critical point method.[13] Elemental analysis of reduction product was carried out with the help of a computer controlled field emission scanning electron microscopy (JEOL JEM-5300) equipped with an energy-dispersive X-ray (EDX) probe to detect Cr and its precipitate compound distribution on and around the cell surface.

### Selection of significant variables by Plackett–Burman

A total of nine independent variables: glucose, (NH_4_)_2_SO_4_, yeast extract, tryptone, KH_2_PO_4_, NaCl, MgSO_4_·7H_2_O, Cr(VI) and inoculum size were used. For the selection of significant variables affecting chromate reduction by *Halomonas* sp. M-Cr, a variety of variables were tested and identified via the Plackett–Burman design experiment.[14] Based on this design, each variable was examined at two levels: −1 for low level and +1 for high level, and a centre point was run to evaluate the linear and curvature effects of the variables. The experimental design with nine factors under investigation with the name, symbol code and actual level of the variables is shown in Table 1. Plackett–Burman experimental design is based on the first-order polynomial model:


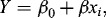


**Table 1.** Plackett–Burman experimental design matrix for evaluation of nine components with the actual and coded levels and the design response for Cr(VI) reduction by *Halomonas* sp. M-Cr.

	Variables/Levels									
	Glucose (g L^−1^)	(NH_4_)_2_ SO_4_ (g L^−1^)	Yeast extract (g L^−1^)	Tryptone (g L^−1^)	KH_2_PO_4_ (g L^−1^)	NaCl (g L^−1^)	Inoculum size (%)	MgSO_4_.7H_2_O (g L^−1^)	Cr(VI) (mg L^−1^)	Chromate reduction (%)
Trials	G	A	Y	Tr	K	N	Is	M	Cr	
1	+1(15)	+1(1.5)	−1(0.25)	+1(5)	+1(0.75)	+1(100)	−1(1)	−1(0.05)	−1(30)	81
2	+1(15)	−1(0.5)	+1(0.75)	+1(5)	+1(0.75)	−1(50)	−1(1)	−1(0.05)	+1(70)	74
3	−1(5)	+1(1.5)	+1(0.75)	+1(5)	−1(0.25)	−1(50)	−1(1)	+1(0.15)	−1(30)	60
4	+1(15)	+1(1.5)	+1(0.75)	−1(1)	−1(0.25)	−1(50)	+1(3)	−1(0.05)	+1(70)	55
5	+1(15)	+1(1.5)	−1(0.25)	−1(1)	−1(0.25)	+1(100)	−1(1)	+1(0.15)	+1(70)	47
6	+1(15)	−1(0.5)	−1(0.25)	−1(1)	+1(0.75)	−1(50)	+1(3)	+1(0.15)	−1(30)	78
7	−1(5)	−1(0.5)	−1(0.25)	+1(5)	−1(0.25)	+1(100)	+1(3)	−1(0.05)	+1(70)	55
8	−1(5)	−1(0.5)	+1(0.75)	−1(1)	+1(0.75)	+1(100)	−1(1)	+1(0.15)	+1(70)	39
9	−1(5)	+1(1.5)	−1(0.25)	+1(5)	+1(0.75)	−1(50)	+1(3)	+1(0.15)	+1(70)	67
10	+1(15)	−1(0.5)	+1(0.75)	+1(5)	−1(0.25)	+1(100)	+1(3)	+1(0.15)	−1(30)	63
11	−1(5)	+1(1.5)	+1(0.75)	−1(1)	+1(0.75)	+1(100)	+1(3)	−1(0.05)	−1(30)	54
12	−1(5)	−1(0.5)	−1(0.25)	−1(1)	−1(0.25)	−1(50)	−1(1)	−1(0.05)	−1(30)	43
13	0(10)	0(1)	0(0.5)	0(3)	0(0.5)	0 (70)	0(2)	0(0.1)	0(50)	60
14	0(10)	0(1)	0(0.5)	0(3)	0(0.5)	0 (70)	0(2)	0(0.1)	0(50)	61

where *Y* is the response (chromate reduction), β_0_ is the model intercept and β*_i_* is the linear coefficient, and *x_i_* is the level of the independent variable. In the present work, 9 assigned variables were screened in 12 experimental runs in addition to 2 runs at their centre point. All trials were performed in duplicate and the averages of chromate reduction results after 24 h were treated as the responses *Y* (Table 1). The results were analysed by the STATISTICA (version 6.0, StatSoft, USA (including parameters estimation and analyses of variance (ANOVA). From the regression analysis of the variables, the factors with significant levels greater than 90% (*P*-value < 0.1) were considered to have a significant effect on chromate reduction.

## Results and discussion

### Screening for chromate reducing alkaliphilic bacteria

While many reports of microbial Cr(VI) reduction are in circulation, very few [15,16] have demonstrated Cr(VI) reduction under alkaline conditions. In addition, high concentration of salts in wastewater treatment systems can be a major problem for conventional biological treatments. Halophilic and alkaliphilic microorganisms produce unique biocatalysts that function under harsh conditions in which their mesophilic counterparts could not survive, permitting the development of additional industrial and bioremediation processes.[5] In this study, 30 unique colonies were selected according to their colony morphology and growth on LB plate amended with 50 mg L^−1^Cr(VI). Preliminary selection of chromate reducing bacteria was estimated qualitatively and quantitatively. A strain designated as M-Cr showed the highest reduction efficiency and was subsequently chosen for further study.

### Characterization of M-Cr

Strain M-Cr formed cream, circular, smooth, bright, mucoid, convex colonies on alkaline LB agar with entire opaque margin, 2–3 mm in diameter. Cells were motile, gram-negative, non-sporulating straight rods, as observed by SEM, with a length of 0.75–1.37 μm and a width of 0.65–1.0 μm (Figure 6A). In addition, the strain M-Cr was oxidase and catalase positive, and best grown in a medium containing up to 20% of NaCl with an optimum at 5% NaCl. The pH growth ranged from 7.0 to 11 with an optimum at pH 9–10.0. According to its growth characteristics, the strain was described as an alkaliphilic, moderate halophilic bacterium.

**Figure 1.**
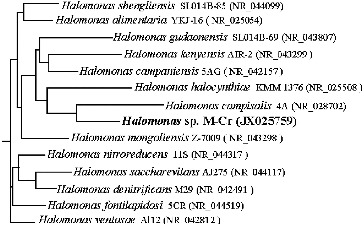
Phylogenetic tree based on 16S rDNA gene sequence, and reference sequences extracted from the GenBank Database, showing the phylogenetic relationship of *Halomonas* sp. M-Cr within representative species of the genus *Halomonas*. Numbers in bracket represents GenBank accession numbers.

**Figure 2.**
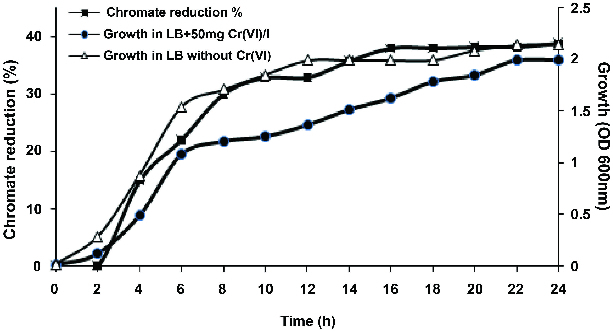
Growth and Cr(VI) reduction efficiency of *Halomonas* sp. M-Cr grown in LB broth, pH 10, in the absence and presence of 50 mg L^−1^ Cr(VI) and incubated at 30 °C under shaking at 120 rpm.

**Figure 3.**
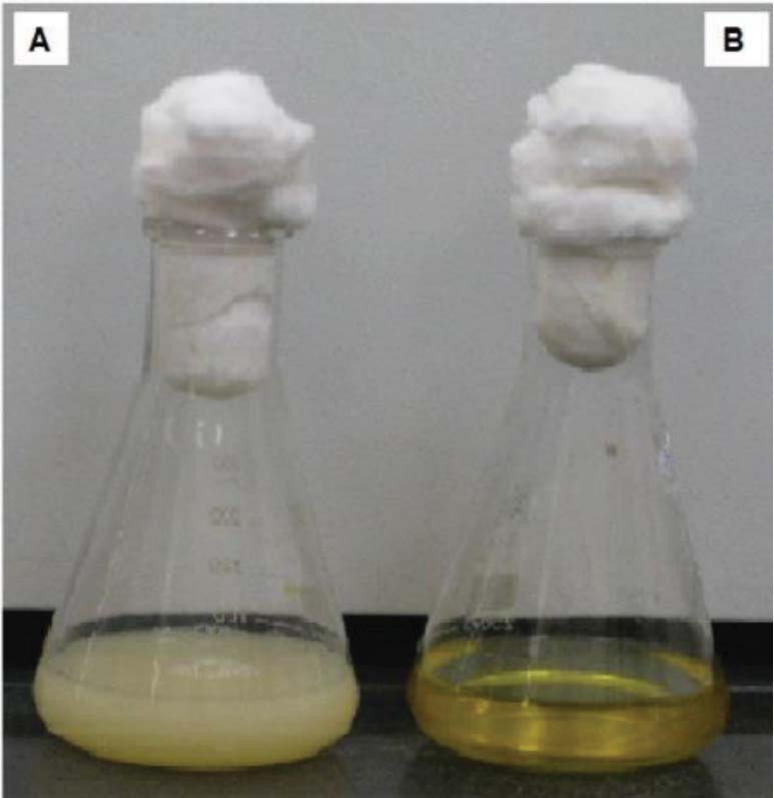
Chromate reduction by *Halomonas* sp. M-Cr. Cells were grown in alkaline LB medium (pH 10) amended with 50 mg L^−1^ Cr(VI), and incubated at 30 °C with shaking at 120 rpm. Complete Cr(VI) reduction was achieved within 120 h and white-precipitate was visible at the bottom of the flask (**A**). Cell-free control was used to monitor any abiotic reduction of Cr(VI) (**B**).

**Figure 4.**
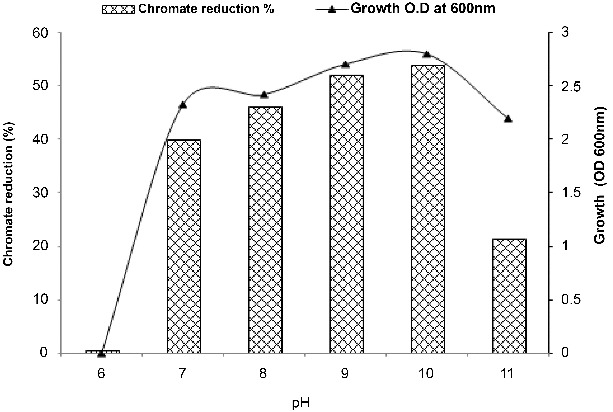
Effect of pH on growth and chromate reduction by *Halomonas* sp. M-Cr after incubation for 48 h with 50 mg L^−1^ Cr(VI), NaCl 0.5%, and agitation of 120 rpm, 30 °C.

**Figure 5.**
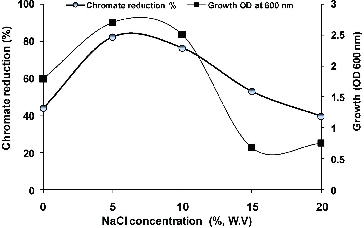
Effect of different concentrations of NaCl on growth and chromate reduction by *Halomonas* sp. M-Cr growing in LB medium of pH 10 with initial Cr(VI) concentration of 50 mg L^−1^ Cr(VI) after incubation for 48 h at 30 °C.

**Figure 6.**
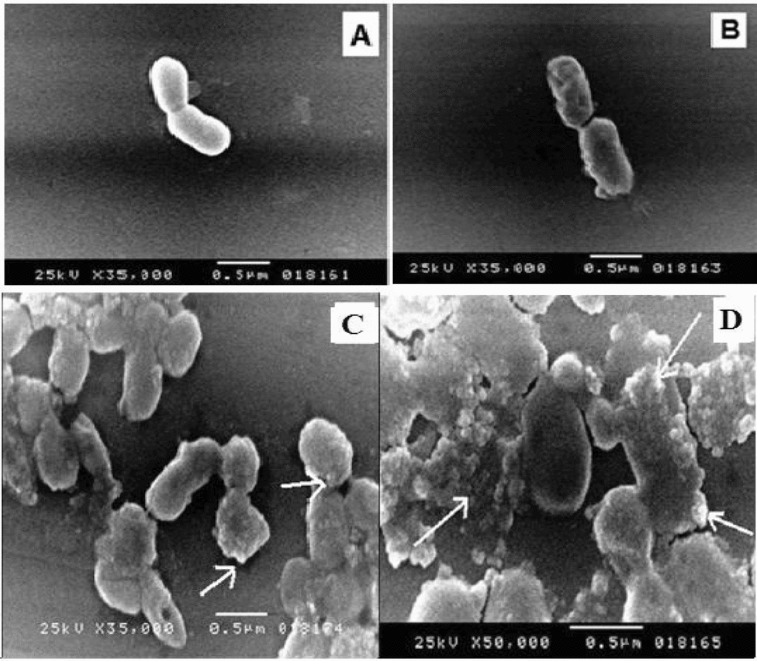
SEM micrographs of *Halomonas* sp. M-Cr cells grown in: (**A**) LB medium without Cr(VI) (control); (**B**) LB medium amended with 50 mg L^−1^ Cr(VI) for 24 h; (**C**) Cr(III) precipitates found as discrete particles bound to the cell surface (arrows); (**D**) Amorphous Cr(III) hydroxide precipitates not attached to cells are also present (arrows), the largest precipitates were slightly rounded.

The 16S rDNA sequence analysis revealed that strain M-Cr shared 91%–99% sequence homology to the species of the genus *Halomonas*, which was included in Phyllum *Proteobacteria* subgroup *Gamma*, in the family *Halomonadaceae*. The 16S rDNA gene sequence was submitted to NCBI GenBank database as *Halomonas* sp. M-Cr with accession number JX025759. The constructed phylogenetic tree (Figure 1) shows that *Halomonas* sp. M-Cr is tightly clustered with *Halomonas campisalis* strain 4A (GenBank accession No. NR028702), isolated from saline lakes in Washington State (USA).[17] Phylogenetic analysis revealed that *Halomonas* sp. M-Cr is included in a group of alkaliphilic, chromate reducers halomonads comprehending *Halomonas mongoliensis* Z-7009 (similarity level 99%), *Halomonas kenyensis* AIR-2 (96%) [18] and *Halomonas campaniensis* 5AG (96%).[19] The identified strain exhibited a high degree of Cr(VI) resistance, being able to grow in the presence of 3500 mg L^−1^ Cr(VI). This was much higher than those of other Cr(VI)-resistant strains: *Halomonas elongate* ATCC33173, *Halomonas subglaciescola* UQM2926 and *Halomonas* sp. TA-04, with values of 50, 125 and 200 mg L^−1^, respectively,[20,21] while it was comparable to other bacterial species, such as *Serratia* sp. Cr-10 (1500 mg L^−1^ Cr(VI)) [22] and *Bacillus* sp. MDS05 (2500 mg L^−1^ Cr(VI)).[23]

### Time course of Cr (VI) reduction by *Halomonas* sp. M-Cr

The growth pattern of *Halomonas* sp. M-Cr in alkaline LB medium (pH 10) containing 50 mg L^−1^ Cr(VI), followed the same growth pattern as without Cr(VI), but with slight inhibition (Figure 2). Reduction was found to be growth associated. No reduction was detected in cell-free medium from the initial to the final stage of the experiment, this indicated no evidence of spontaneous Cr(VI) reduction and the major mechanism of reduction was attributable to microbial metabolism.[24] Also the formation of precipitates only in cultures supplemented with Cr(VI) illustrated the transformation of this soluble oxyanion to Cr(III) that forms insoluble hydroxide Cr(OH)_3_ as a white precipitate (Figure 3). Comparing chromate reduction between different studies can be difficult, given the wide range of culturing conditions used and the effect such conditions can have on reduction process.[25] Under alkaline conditions, *H*. *chromatireducens* AGD 8-3 was able to reduce 80% of 5 mg Cr(VI)/l, while *H. campisalis* Z-7398, *H. desiderata* FB2, *H. kenyensis* AIR-2, *H. natronophila* Z-7009 and *H. campaniensis* 5AG reduced approximately 50%, 35%, 25%, 25% and 5% of chromate, respectively.[26] *Halomonas* sp. MV-2007, reduced about 75% of the 5 mg L^−1^ Cr(VI) after 25 days.[16]

### Effect of pH and salinity on *Halomonas* sp. M-Cr growth and Cr (VI) reduction

Data in Figure 4 confirm that *Halomonas* sp. M-Cr cells favour the reduction under alkaline conditions compared to neutral or acidic conditions. The first report on Cr(VI) reduction under alkaline conditions by Halomonads was reported by Van Engelen et al. [16] for *H*. sp. MV-2007, isolated from Soap Lake, a chemical stratified alkaline lake located in central Washington State, USA, followed by Shapovalova et al. [26] for *H*. *chromatireducens* AGD 8-3.

As depicted in Figure 5, maximum growth and chromate reduction (82.22%) were observed in the presence of 5% NaCl after 48 h incubation. Regarding the genus *Halomonas*, only *H. chromatireducens*, isolated from soda solonchak soils of the Kulunda steppe (Russia) and *H*. sp. TA-04, isolated from polluted marine sediments near a stainless steel plant in Southern Italy have been described as a Cr(VI) reducer under high salinity.[21,26]

### SEM and EDX analysis of *Halomonas* sp. M-Cr cells

Trivalent form of chromium is known to readily precipitate as chromium hydroxide [Cr(OH)_3_] above pH of 5.0.[27] Cell morphology of *Halomonas* sp. M-Cr was observed with SEM after cultivation of bacteria for 48 h with and without Cr(VI). In the presence of Cr(VI), elongation of cells (2.0–3.0 μm) with appearance of wrinkles on the surface was observed (Figure 6B). Similar effects on cellular morphology and surface topology has been observed in other gram-negative bacteria like: *Acinetobacter haemolyticus* and *Serratia* sp. Cr-10.[22,28] Also SEM analysis revealed some bacterial cells encrusted with amorphous precipitates or the precipitate was formed at random sites (Figure 6C, Figure 6D). The largest precipitates were slightly rounded. Similar results were obtained for *Shewanella oneidensis* and *Acinetobacter haemolyticus* [29,30] in which various sizes of Cr(III) precipitates were bound to the cell and restricted to the outer surface after chromate reduction. The precipitate was assumed to be in the Cr(III) form, Cr(OH)_3_, due to the inability of chromate anions to bind with electronegative surface functional groups (e.g. carboxyl, phosphoryl and hydroxyl) commonly found on gram-negative envelopes.[28,31]

**Figure 7.**
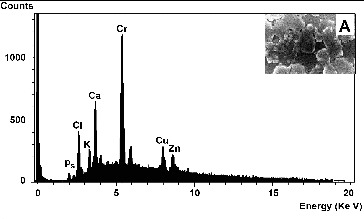
EDX spectrum analysis of amorphous precipitates that surrounded *Halomonas* sp. M-Cr cell surfaces during Cr(VI) reduction (**A**). An EDX spectrum from the dense particles generated a large Cr peak, indicating that it is most likely an amorphous Cr(III) hydroxide. Inset: SEM images of *Halomonas* sp. M-Cr cells and precipitates. The whole area was analysed with EDX.

Elemental analysis of the amorphous precipitate by EDX revealed that the peak corresponding to chromium was higher than for other elements (Figure 7), indicating that Cr was the major element comprising 46.3% of the total weight of the precipitate. The SEM image (Figure 7, inset A) shows the presence of Cr(OH)_3_ precipitates adhered to the surface of the rods shaped cells.

### Screening of significant variables using Plackett–Burman design

To the best of our knowledge, very few studies applied statistical design to optimize bioreduction of Cr(VI).[32–35] Plackett–Burman design, an efficient technique for medium component optimization,[35,36] was employed to identify significant variables that enhance chromate reduction by *Halomonas* sp. M-Cr and to find out their probable optimal levels in a limited number of experiments. Nine variables were analysed with regard to their effects on chromate reduction using a Plackett–Burman design. The responses in Table 1 show a wide variation in Cr(VI) reduction efficiency, ranging from 39% to 81% corresponding to the combined effect of the nine parameters in their specific ranges.

Analysis of variance (ANOVA) for the results of Plackett–Burman design is shown at Table 2. The model determination coefficient of the regression model (*R* ^2^ = 0.9669) indicates that 96.69% of the variability in the response could be explained by the model. Adjusted determination coefficient (Adj *R* ^2^ = 0.8179) was also high in order to support a high significance of the model.

**Table 2.** Statistical analysis of Plackett–Burman design results.

Variables	Coefficient	Effect	*t-*value	*P*-value	Confidence level (%)
Intercept	59.71	–	–	–	–
Glucose	6.71	13.42	4.02	0.06	94
(NH_4_)_2_SO_4_	1.04	2.08	0.62	0.6	40
Yeast extract	−2.18	−4.36	−1.31	0.32	68
Tryptone	7.015	14.03	4.21	0.05	95
KH_2_PO_4_	5.85	11.7	3.51	0.07	93
MgSO_4_·7H_2_O	−0.71	−1.42	−0.42	0.71	29
NaCl	−3.15	−6.3	−1.89	0. 2	80
K_2_CrO_4_	−3.52	−7.04	−2.11	0.17	83
Inoculum size	2.32	4.64	1.39	0.3	70

Note: *R* ^2^ = 0.9669; Adj *R* ^2^ = 0.8179.

**Figure 8.**
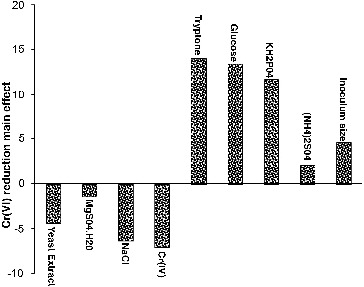
Positive and negative influence of different variables on Cr(VI) reduction by *Halomonas* sp. M-Cr based on the result of Plackett–Burman design.

Main effect analysis revealed that, five out of the nine variables (glucose, tryptone, KH_2_PO_4_, (NH_4_)_2_SO_4_ and inoculum size) included in this study were found to have a positive influence on Cr(VI) reduction, indicating that the higher concentrations of these variables are ideal for enhancing Cr(VI) reduction, whereas chromate, yeast extract, NaCl and MgSO_4_·7H_2_O had negative effect towards reduction process, indicating that lower concentrations of these factors in experimental range were favourable for increasing Cr(VI) reduction (Figure 8). Variables with the confidence levels greater than 90% were considered as significant. Tryptone was considered the most significant factor (95% confidence level), followed by glucose at 94%, and KH_2_PO_4_ at 93%. The confidence levels of other variables were below 90%; hence, their individual effects were negligible. If the variables which were insignificant were to be neglected, the model equation for chromate reduction efficiency can be written as

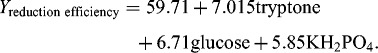

The positive correlation between tryptone concentration and chromate reduction implies that a higher concentration is more effective in increasing reduction efficiency in the chosen experimental limits. This indicates nutrient requirement for optimum chromium reduction which depends on the nature of microbial employed species.[34] The positive significance effect of glucose on reduction process by *Halomonas* sp. M-Cr was probably due to the increase in metabolic activity. It has been found that chromium transport into the bacterial cell depends on energy. Therefore, it is a glucose dependent process.[37] These results are in consistence with other reports indicating requirement of glucose as electron donor for Cr(VI) reduction.[5,21,35,38] Phosphate sources play a crucial role in cellular respiration and metabolism of the microbes which induces the microbe to uptake the metal ions.[39]

### Verification of the predicted optimum variables

The Plackett–Burman design predicted that the higher chromate reduction efficiency was achieved in a medium containing (g L^−1^): glucose 15, (NH_4_)_2_SO_4_ 1.5, yeast extract 0.25, tryptone 5, KH_2_PO_4_ 0.75, MgSO_4_·7H_2_O 0.05, NaCl 50 and pH 10.0. In order to evaluate the accuracy of the applied Plackett–Burman design, a verification experiment was carried out in triplicate. The average of reduction of predicted near optimum levels of independent variables were examined and compared to the centre condition settings. Optimization improved the Cr(VI) reduction efficiency to 100% of 50 mg L^−1^ Cr(VI) in 12 h compared with 60% removal in 24 h before optimization, which represented an increase in reduction efficiency of 40%. These results confirm the validity of the optimized medium.

## Conclusions

This study reports the isolation of a potent Cr(VI) reducing moderate halophilic and alkaliphilic *Halomonas* sp. M-Cr from tannery effluent. Potential reduction of chromium by *Halomonas* sp. M-Cr was optimized by employing Plackett–Burman design. This design helped in locating the optimum levels of the most significant parameters which contribute to the maximum Cr(VI) reduction. Plackett–Burman not only demonstrated the increase in chromate reduction by *Halomonas* sp. M-Cr at the optimized conditions but also proved to be simple, efficient and time and material saving. The ability of the strain to reduce Cr(VI) in the presence of high salinity as well as at high pH expands the opportunities for bioremediation of marine polluted environments or wastewaters containing high salt concentrations. To the best of our knowledge, this is the only report available in the literature on chromium reduction by *Halomonas* sp. isolated from tannery effluents.
